# An assessment of natural product discovery from marine (*sensu strictu*) and marine-derived fungi

**DOI:** 10.1080/21501203.2014.931308

**Published:** 2014-07-16

**Authors:** David P. Overy, Paul Bayman, Russell G. Kerr, Gerald F. Bills

**Affiliations:** ^a^ Department of Chemistry, University of Prince Edward Island, 550 University Ave., Charlottetown, Prince Edward Island, CanadaC1A 4P3; ^b^ Department of Pathology and Microbiology, Atlantic Veterinary College, University of Prince Edward Island, 550 University Ave., Charlottetown, Prince Edward Island, CanadaC1A 4P3; ^c^ Department of Biomedical Sciences, Atlantic Veterinary College, University of Prince Edward Island, 550 University Ave., Charlottetown, Prince Edward Island, CanadaC1A 4P3; ^d^ Nautilus Biosciences Canada, Duffy Research Center, University of Prince Edward Island, 550 University Ave., Charlottetown, Prince Edward Island, CanadaC1A 4P3; ^e^ Department of Biology, University of Puerto Rico-Río Piedras, P. O. Box 23360, San Juan00931, Puerto Rico; ^f^ Texas Therapeutics Institute, The Brown Foundation Institute of Molecular Medicine, University of Texas Health Science Center, 1881 East Rd., Houston, TX77054, USA

**Keywords:** marine biodiversity, marine mycology, osmotolerance, secondary metabolites

## Abstract

The natural products community has been investigating secondary metabolites from marine fungi for several decades, but when one attempts to search for validated reports of new natural products from marine fungi, one encounters a literature saturated with reports from ‘marine-derived’ fungi. Of the 1000+ metabolites that have been characterized to date, only approximately 80 of these have been isolated from species from exclusively marine lineages. These metabolites are summarized here along with the lifestyle and habitats of their producing organisms. Furthermore, we address some of the reasons for the apparent disconnect between the stated objectives of discovering new chemistry from marine organisms and the apparent neglect of the truly exceptional obligate marine fungi. We also offer suggestions on how to reinvigorate enthusiasm for marine natural products discovery from fungi from exclusive marine lineages and highlight the need for critically assessing the role of apparently terrestrial fungi in the marine environment.

## Introduction

**Figure 1.**
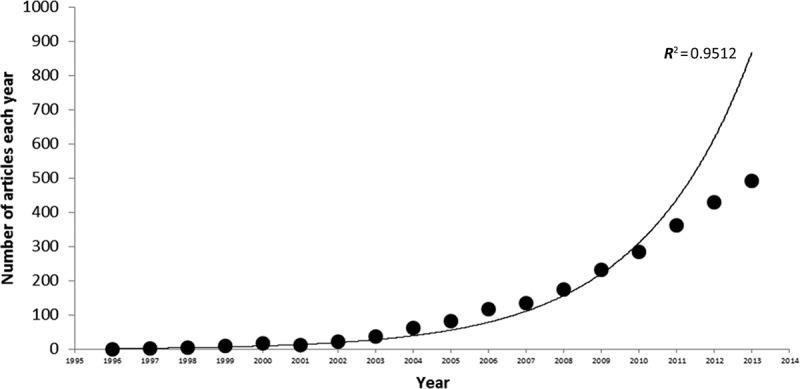
Number of articles from 1996 until April 2014 that include either ‘marine-derived fungus’ or ‘sponge-derived fungus’ in title, abstract, or keywords. An exponential trend line was fitted to the actual data points (*R* ^2^ = 0.95 if 2014 is excluded). Data were extracted from Scopus on 15 April 2014.

The term *marine-derived fungus* can be traced back to the 1990s (Wang et al. 1997; Christophersen et al. 1998). During the last 15 years, an avalanche of papers (Figure 1) have appeared describing fungal metabolites from strains of fungi isolated from oceans, ocean-dwelling animals, marine algae, shorelines and estuaries, and from marine–terrestrial transitional habitats, e.g., mangroves and other halophytes. In nearly all cases, these fungi were not species that belong to the well-documented lineages of marine fungi, or in other cases, the organism was not definitively identified; they have been called ‘marine-derived.’ In principal, the term ‘marine-derived’ simply indicates location in the marine environment. Unfortunately, in some cases, the word ‘marine’ appears to have been attached to isolates to justify that the research had accomplished a marine-specific objective or that the metabolite-producing organism might be different from a genotypically related terrestrial counterpart.

Progress in revealing the extent of fungal biosynthetic capacity is welcome, and the interest in fungi from the marine environment has significantly expanded the catalog of fungal products including many novel chemical scaffolds. The range of chemistry found in marine-derived fungi has been reviewed numerous times during recent years (Bugni & Ireland 2004; Saleem et al. 2007; Rateb & Ebel 2011). One metabolite, halimide, a potently cytotoxic diketopiperazine phenylahistin from a marine-derived *Aspergillus ustus* led to the synthesis of plinabulin (NPI-2358). Plinabulin entered phase II trials as an angiogenesis inhibitor for preventing tumors and scar tissue development (Mita et al. 2010). But, rarely in these reports do the authors question whether their metabolite-producing organisms truly depend on the marine environment or whether genotypically related strains of the same species from a terrestrial habitat might produce the same compounds under identical fermentation conditions (Capon et al. 2005). Although, these marine-derived strains are promoted as a new and unexplored source of chemistry, few comparative data exist to test the hypothesis that a search of other underexplored ascomycete or basidiomycete fungi would result in an equal, if not better, probability of encountering novel biosynthetic gene clusters and associated chemistry (Dreyfuss 1986; Dreyfuss & Chapela 1994; Hosoya 1998; Okuda et al. 2005; Gloer 2007; Bills et al. 2013).

A puzzling aspect of this accelerated interest in ‘marine-derived’ fungi is that many chemists, chemical ecologists, and other scientists interested in the application and function of natural products in chemical biology, and who would generally take a rigorous approach when exploring plant and animal natural products and their relationships to their producing organisms, apparently relax this rigor when it comes to marine microorganisms in general, and to fungi specifically. Isolation of a fungal culture from a propagule embedded in a matrix does not necessarily mean the fungus depends on the matrix as its habitat. For fungi, dependence on the habitat generally is proven when the organism’s vegetative state can be shown to colonize and interact with its substratum and that the organism disperses, via spores or vegetative propagules, back to its substratum to reinitiate its life cycle. Of course, fungi vary enormously in the breadth and fidelity of their substratum relationships. Nevertheless, examination of the lists of marine-derived fungi indicates that they are often taxa with ruderal substratum relationships, and fungi that are often characteristic of soils and plant surfaces are among the most frequently cited. For example, one can isolate fungi from the air, and the biodiversity of airborne fungal propagules based on both metagenomic and direct sampling data is astonishing (Fröhlich-Nowoisky et al. 2009, 2011; Amend et al. 2010); however, as far as we know, no one has proposed that fungi colonize the atmosphere, nor has anyone published research regarding the discovery of novel chemistry from atmosphere-derived fungi.

Taking into consideration the interest, activity, and resources being expended on exploring marine-derived fungi, we asked the question of whether a concurrent increase in the knowledge of secondary metabolism from acknowledged lineages of marine fungi has resulted. Surprisingly, based on our review of the literature (Figure 1), summarized below, the rate of discovery from acknowledged marine fungi may have decreased since the late 1990s, or at best remained flat. Below, we review the modest amount of available information on the chemistry of fungi from exclusively marine lineages. The phylogenetic breadth of marine fungi is great, including marine-specific yeasts, chytrids, and other basal fungal groups. However, this review will focus on filamentous ascomycete and basidiomycete fungi from the marine environment, as fungi in the Dikarya are those most likely to have the genes necessary for secondary metabolite biosynthesis (Arvas et al. 2007; Lackner et al. 2012; Ohm et al. 2012). Our intention is not to question whether marine-derived fungi are worthy of intensive exploration. Rather, we examine some of the reasons for the apparent neglect of the truly exceptional obligate marine fungi, and suggest some ways to redirect the energy expended on the low-hanging fruit of marine-derived fungi towards the challenges of investigation of marine fungi (*sensu strictu*).

## What is a marine fungus?

The branding and classification of all fungal isolates obtained from marine sources as being of marine origin continues to be debated (Table 1). Labeled as ‘marine-derived’ fungi by the natural products community, their relationship to the marine habitat is ambiguous. The opinion voiced by the mycological community and some natural products chemists is that the majority of marine-derived fungi reported in the natural products literature likely come from terrestrial habitats (Höller et al. 2000; Jensen & Fenical 2002; Kohlmeyer & Volkmann-Kohlmeyer 2003a), where dormant propagules (i.e., spores) are randomly transported to the sea by air and water, where they persist until they are discovered. Classification of a fungus as being of marine origin requires that the fungus plays an active role in the marine environment. Unlike certain kinds of marine bacteria (Jensen & Fenical 2005), marine fungi cannot be defined solely on physiological criteria (i.e., requirement of salt for growth), as vegetative growth does not require the presence of seawater (although maximum growth typically occurs at 20–60% seawater; (Jones & Jennings 1964)) nor is seawater required to induce sporulation for all marine fungi (although some require salinities of 40–100% seawater (Byrne & Jones 1975)). Rather, the classical definition of ‘marine’ is based more on the ecology of the organism where marine fungi are classified into obligate and facultative forms: obligate marine fungi are those that grow and sporulate exclusively in a marine or estuarine habitat, and facultative marine fungi are those that also occur in freshwater or terrestrial milieus yet are able to grow in, and frequently are isolated from marine habitats (Kohlmeyer & Kohlmeyer 1979). The issue is complicated by the fact that there are several partially overlapping definitions based on different criteria (Table 1).

**Table 1.** Definitions of marine fungi *sensu latu.*

Term	Level	Criterion	Definition	Example
Marine (*sensu strictu*) vs. marine-derived	Strain	Physiological	Active growth and sporulation in a marine substratum	Jones et al. (2009)
	Strain	Substrate	Isolated from a marine substratum	Wang et al. (1997)
Obligate marine vs. facultative marine	Species	Habitat	Grow and sporulate exclusively in a marine or estuarine habitat	Kohlmeyer (1981)
	Species	Habitat	Grow in the marine environment but originate from freshwater or terrestrial milieus	Kohlmeyer (1981)
Primary marine vs. secondary marine	Genus or above	Phylogeny	From a lineage which evolved in the sea	Kohlmeyer (1986)
	Genus or above	Phylogeny	From a lineage which recently colonized the sea	Kohlmeyer (1986)

Unfortunately, without measurements of vegetative growth in the sediment, discriminating an actively growing sediment saprobe from the isolation of a spurious propagule is difficult. Spores of terrestrial fungi, carried to the sea by wind or rain, may remain viable for some time without germinating. Most isolation techniques do not distinguish between fungi actively growing in a substratum and ungerminated spores casually associated with it; both may grow when sown in culture media. Several techniques can discriminate between active growth and spurious propagules, but all are difficult. Ergosterol, a sterol unique to fungal cell membranes, can be used as a biomarker for fungal growth (Gessner & Chauvet 1993). Another approach is to detect secondary metabolites *in situ*. In one such study, several *Trichoderma* strains were isolated from sediment samples taken from the French Atlantic coast and were confirmed by liquid chromatography-tandem mass spectrometry (LCMS/MS) analysis to produce longibrachins and trichokonins (20-residue peptaibols) in culture (Poirier et al. 2007). LCMS/MS analysis of the original sediment samples demonstrated the presence of the aforementioned longibrachins and trichokonins, in a sufficient concentration (5.2 ± 2.1 ng/g of sediment) to confirm the occurrence of active growth of fungi within the sediment (Poirier et al. 2007). A third approach is to detect fungal gene expression in the substratum using rtPCR or metatranscriptomes (Edgcomb et al. 2011; Burgaud et al. 2013). Studies like these can demonstrate the growth of fungi in marine substrata, and will be required before many ubiquitous species are generally accepted as being facultative marine fungi by the mycological community.

Recently, the long-standing Kohlmeyer definition of what constitutes a marine fungus has been challenged. In the latest review on marine mycology, Jones et al. (2009) stated that whether or not a fungus is obligatorily or facultatively marine depends largely on personal opinion, especially regarding the facultative status of ubiquitous saprobic species isolated from marine sediments and from intertidal substrata such as maritime grasses and mangroves. Fungi isolated from marine sediments have been largely ignored by marine mycologists (Kohlmeyer & Volkmann-Kohlmeyer 2003a) and as a result are typically labeled as terrestrial. On the other hand, the repeated isolation of these species from such habitats has some marine mycologists arguing for a reevaluation of their status (Jones et al. 2009). However, having multiple terms (i.e., terrestrial, facultative marine, etc.) to describe in part the physical location of where a propagule of a species is encountered, without knowledge that the mycelium is established, leads to confusion. The definition of ‘facultative marine’ encompasses fungi from terrestrial and freshwater environments that have also been observed to grow in the marine environment. A ubiquitous species, by definition of the word ‘ubiquitous’, is a species whose viable propagules are found to occur everywhere. Here we employ the term ‘marine (*sensu strictu*)’, to define those fungi that exclusively carry out their life cycles in marine or estuarine habitats (synonymous with ‘obligate marine’), and ubiquitous, to define those fungi whose propagules can establish mycelium and successfully disperse to establish a new generation in both terrestrial and marine habitats. Adopting the term marine (*sensu strictu*) will redirect the current confusion regarding whether or not a well-known terrestrial strain should be considered as marine.

## The osmophilic nature of many terrestrial fungi confounds the question

Rarely is the osmophilic nature of marine-derived fungi investigated in relation to their metabolite expression (Huang et al. 2011; Wang et al. 2011). It is likely in many cases that these fungi are not necessarily only salt-tolerant, but rather osmotolerant (Pitt & Hocking 1997). Such fungi are adapted to grow in low-water-activity habitats and likely would grow equally well in elevated concentrations of sugars or glycerol, and in inland saline environments (Jin et al. 2005; Gal-Hemed et al. 2011). Examination of species lists of marine-derived fungi indicates a dominance of typical osmotolerant genera (e.g., *Penicillium, Aspergillus, Phoma, Alternaria, Scopulariopsis*, and many others) and mirror species lists isolated from hypersaline environments (Anastasiou 1963; Buchalo et al. 1998; Kis-Papo et al. 2003; Cantrell et al. 2006; Walker & Campbell 2010). A question can be raised related to the use of seawater and artificial seawater supplements in media formulations: does the presence of salt effect the perceived distinctiveness of the chemistry of marine-derived isolates relative to their terrestrial counterparts? For example, a group working on the chemistry of a terrestrial isolate of *Aspergillus terreus* would be unlikely to supplement the medium with artificial or natural seawater, although they might elect to grow the fungus in a medium with high solute concentrations. A group working with a marine isolate of *A. terreus* would almost certainly supplement the medium with sea salts; if a silent pathway was activated due to high sodium or halide concentrations or increased osmotic stress, the discovery of that new metabolite would likely be attributed to the marine origin of the fungal isolate. An example of this effect was observed during investigation of extracts of a soil strain of *Aspergillus unilateralis* that were toxic to *Saccharomyces cerevisiae*, a NS-1 cell line, *Bacillus subtilis* and the nematode, *Haemonchus contortus* (Capon et al. 2005). Among its many metabolites, this fungus produced the dipeptide penicillazine, first reported from a marine-derived *Penicillium* sp. The same compound has also been called trichodermamide A when it was isolated from a marine isolate of *Trichoderma virens*. During scale-up for purification of *A. unilateralis*, 1% NaCl was added to the solid wheat culture resulting in co-expression and the appearance of trichodermamide B, previously described from same marine isolate of *T. virens. Aspergillus insulicola* is a species characteristic of intertidal areas, yet terrestrial and marine-influenced isolates exhibited consistent metabolite profiles that included penicillic acid, 4-hydroxymellein, xanthomegnin, viomellein, vioxanthin, and asteltoxin (Rahbæk et al. 1997). The nitrobenzoyloxy-substituted sesquiterpene, insulicolide A could be produced on both Czapek-yeast autolysate medium and soybean–starch seawater medium, but not on a medium with high sucrose concentrations. In both cases, the authors concluded that environmental factors, including solutes and media components, were more influential in affecting metabolite expression profiles than genetic factors.

Experimental methods are available to test the hypothesis that pathways might be differentially activated in a terrestrial isolate grown under saline conditions. The addition of sea salt or any other solute is likely to profoundly affect secondary metabolism because of the interconnectivity of the osmotic stress regulatory system and fungal development (Jin et al. 2005; Duran et al. 2010; Wang et al. 2011). In the case of osmotically adapted fungi, for example, aspergili and penicillia, increasing solute concentrations may positively affect growth and asexual sporulation. In both yeasts and filamentous fungi, the process of osmotic adaptation by activation of the HOG pathway results in the biosynthesis and accumulation of compatible molecules such as proline, trehalose, polyols, and glycerol to counterbalance the osmotic pressure and prevent water loss (Duran et al. 2010). Thus, the contributions of solute tolerance and marine adaptation to secondary marine metabolite discovery are confounded. On the other hand, the marine environment obviously would be a logical place to search for halotolerant fungi.

## A case study: *Aspergillus*

**Figure 2.**
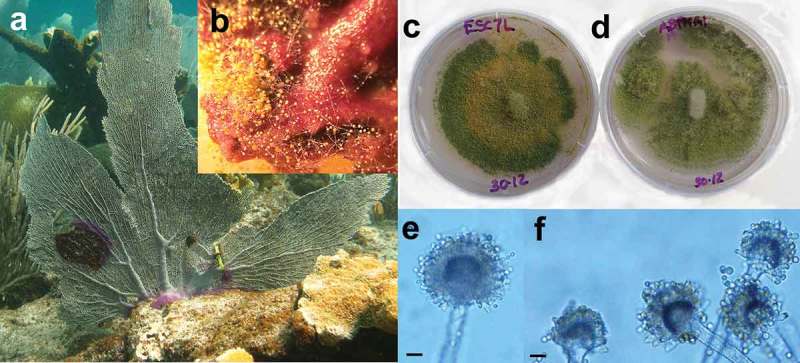
Marine-derived and terrestrial strains of *Aspergillus flavus*. (a) Colony of the sea fan *Gorgonia ventalina* showing purple lesions, called ‘aspergillosis’ in the literature. (b) Surface-sterilized piece of sea fan tissue plated on agar. *Aspergillus flavus* is growing out, as is common in both diseased and healthy tissue. (c) and (d) strains of *A. flavus* from sea fan tissue and terrestrial substrata, respectively. (e) and (f) Conidiophores of the *A. flavus* strains in (c) and (d). Scale bars = 10 µm. Photo in (a) by Anabella Zuluaga-Montero. © [Anabella Zuluaga-Montero]. Reproduced by permission of Anabella Zuluaga-Montero.

An example of active participation of ubiquitous fungi in the marine environment comes from *Aspergillus* species associated with the sea fan coral *Gorgonia ventalina*. The pathogen of sea fan aspergillosis was reported to be *A. sydowii*, mainly known as a terrestrial fungus but also isolated from marine substrata (Geiser et al. 1998). However, *A. sydowii* was found to be far more common in healthy than in diseased sea fan tissue, calling its pathogenicity into question (Toledo-Hernández et al. 2008). *Aspergillus flavus* was far more common than *A. sydowii* in diseased sea fan tissue (Figure 2), suggesting it may be an opportunistic pathogen in sea fans, as it is in humans.

Thus, a key question is whether *A. sydowii* and *A. flavus* are composed of distinct marine and terrestrial ecotypes, genetically isolated from each other, or whether the same populations are able to colonize both land and sea? In the context of sea fan disease, this question is important because there is considerable interest in identifying the source of inoculum. For example, African dust clouds were proposed as the source of the pathogen in the Caribbean (Shinn et al. 2000; Weir-Brush et al. 2004), which assumes that terrestrial and marine populations do not differ. Adaptation to marine habitats has happened in diverse lineages of fungi, as phylogenetic studies show (Spatafora et al. 1998; Hibbett & Binder 2001). However, in the case of *Aspergillus*, the available evidence points to single populations in both land and sea: DNA fingerprinting studies in *A. sydowii* using microsatellites (Rypien et al. 2008) and in *A. flavus* using AFLPs (Zuluaga-Montero et al. 2010; Ramírez-Camejo et al. 2012) concluded that these species are not differentiated into terrestrial and marine clades. Furthermore, marine and terrestrial isolates did not differ in virulence when inoculated in *Drosophila* flies (Ramírez-Camejo et al. 2014). These studies establish that *A. flavus* and *A. sydowii* are neither obligate marine nor primary marine fungi (in the sense of Table 1). At least for these two ubiquitous, ruderal species, the marine–terrestrial boundary appears to be of limited consequence.

The aspergilli and penicillia offer the most accessible models for dissecting questions about marine-derived fungi (see comments below regarding work on marine Actinomycetes). Chemometric, genetic, and now comparative genomic methods are well established for the detection of metabolites and for mapping of many already functionally characterized biosynthetic pathways. Reference strains are abundant in culture collections. The large number of reports of metabolites from marine-derived penicillia and aspergilli would more than justify in-depth comparative investigations to map the extent of genetic isolation in the marine environment, and whether this has led to genetic drift in common secondary metabolite genes, or whether the marine environment favors acquisition of new gene clusters through horizontal gene transfer. Finally, extensive monographic works, multigene phylogenies, and chemotaxonomic data are available for most major groups of the Trichocomaceae; therefore, there is little reason not to attempt a thorough identification of marine-derived strains from these genera.

## Biodiversity of marine fungi

Marine mycology has grown into a significant mycological subdiscipline during the last century with mycologists reporting the occurrence of marine fungi as early as 1849 (Jones et al. 2009). By 1996, diversity estimates of marine fungi were placed at around 1500 species, and by 2011, estimates projected the number of possible marine fungi as over 10,000 species (Jones 2011). However, this may be an overestimate as it is inclusive of many ubiquitous species (i.e., facultative and ‘marine-derived’ species). A total of 530 filamentous fungi have been described to date from marine sources (Jones et al. 2009) and are considered here under the definition of marine (*sensu strictu*). As new substrata and geographical locations are examined for fungi, the number of total species is expected to increase through the continued discovery of new species. The distribution of the fungi reported from marine sources can be classified as follows: Ascomycota 424 species, anamorphic fungi 94 species, and Basidiomycota 12 species, with Halosphaeriales being the largest order represented by 126 species (Jones et al. 2009). Inclusive within the orders of Ascomycetes only taxa of the Halosphaeriales and Lulworthiales consist only of aquatic fungi (predominantly marine species) (Kohlmeyer et al. 2000; Sakayaroj et al. 2011). According to Jones et al. (2009), classification of many marine fungi remains a confused and unresolved issue, and is particularly acute for the Ascomycota, as 70 of the genera are referred to as taxa *incertae sedis*.

## Where do marine fungi thrive?

Fungi are major decomposers of woody and herbaceous substrata entering marine ecosystems. Ecological habitats of marine fungi include submerged wood, mangroves, sands, sediment, algae, estuary plants, invertebrates, plankton, and probably even the plastisphere (Zettler et al. 2013). Surprisingly, we still lack a good understanding of fungi in deep sea sediments, but a few analyses of clone libraries constructed from DNA and cDNA already indicate that fungi appear to be the dominant eukaryotes of deep sea subsurface sediments, and the subsurface is particularly rich in single-celled forms of the Basidiomycetes and Ustilaginomycetes (Edgcomb et al. 2011). In contrast, fungal diversity recovered by culture-dependent methods in the deep sea indicated that many are phylogenetically related to terrestrial fungi. As pointed out by Burgaud et al. (2013), carefully planned diversity assessment employing molecular, microscopic, and cultivation approaches to what constitutes a marine fungus and whether these fungi are active in the subsurface or whether these organisms are merely preserved as inactive propagules are needed to accurately interpret the significance of different fungal groups in marine subsurface samples.

Submerged wood substrata tend to favor the growth of members of the Halosphaeriales and Lulworthiales, especially in open oceans where species are typically characterized by passive ascospore release (deliquescing asci) and appendaged ascospores that aid in floatation and attachment (Jones 1995). Mangrove ecosystems produce abundant plant detritus and faunal biomass. Over 280 species of fungi have been described from submerged mangrove substrata (Jones 2011). These fungi are often found on driftwood in the intertidal zone, often extruding spores during periods of low tide, where ascospores generally are found to have gelatinous sheaths rather than appendages. Arenicolous marine fungi have adapted to sporulate on sand grains or other hard surfaces as ascomata lack long central necks (that would be abraded by the constant movement of the sand grains) and ascospores are appendaged and often found to be trapped in sea foam (Kohlmeyer & Kohlmeyer 1979; Jones 2011). Although algal flora dominate marine habitats in temperate regions, relatively few algae have been examined in detail for associated fungi; however, approximately 80 fungal species have been associated with algae either as parasites or symbionts (Zuccaro & Mitchell 2005; Jones 2011). The estimated number of marine algae currently ranges between 9500 and 12,500 species (Jones 2011). Thus, the number of currently associated fungal species is an extremely low estimate of the total number of probable associated species, and further isolation efforts are warranted. Estuarine (or salt) marshes occur in the intertidal zone and are dominated by the growth of salt-tolerant plants. Fungi associated with *Spartina* species are numerous and have been well studied because of the plant’s dominant role in estuaries and the proximity of estuaries to major universities (Gessner & Goos 1973; Eleuterius & Meyers 1977; Newell et al. 1996; Pazoutova et al. 2000; Amend et al. 2010; Walker & Campbell 2010; Elmer & Marra 2011). Despite this surprising species richness, very few studies of fungi associated with herbaceous estuarine marsh plants have been undertaken. For example, from studies of one sea monocot alone (*Juncus roemerianus*), 107 fungal species were isolated: 44 obligate marine species, 25 facultative marine species, and 38 halotolerant terrestrial species (Kohlmeyer & Volkmann-Kohlmeyer 2001). Extrapolation to other marine plants therefore suggests a tremendous opportunity for the discovery of new fungal species.

Reports of marine fungi (*sensu strictu*) obtained from invertebrates in the literature are rare and include approximately 20 species (Kohlmeyer & Volkmann-Kohlmeyer 2003b). In addition to certain aspergilli that have been implicated in coral decline, hyphae of yet uncharacterized fungi have been observed to be associated with mollusk shells and other carbonate animal remains, where they are believed to be involved in perforation of mineralized tissue (Golubic et al. 2005). Such fungi are often difficult to culture, and visual confirmation of growth *in situ* is one factor hindering the discovery of more of these fungi. Their investigation may lead to the recognition of new and widespread types of marine fungi (Golubic et al. 2005; Liñan-Rico & Cardenas-Conejo 2013).

## Trends in natural products discovered from marine fungi (*sensu strictu*) and marine-derived fungi

Based on literature reviews encompassing the period from 1970 to 2010, the rate of discovery of new natural products from marine and ‘marine-derived’ fungi has increased exponentially (Figure 1). During 1970–2002, a total of 272 natural products were characterized from 80 fungal isolates originating from marine substrata; predominantly algae (27%) and sponges (28%) (Bugni and Ireland 2004). Of these isolates, only 16 represented species regarded as marine fungi (*sensu strictu*), from which chemical investigations yielded the discovery of 41 natural products. The remaining 64 isolates could be classified into two groups: 30 isolates representing ubiquitous fungal species and 34 which were unidentified (or identified only to the generic level) preventing their classification as marine fungi. A dramatic increase in the rate of discovery of natural products from marine-derived fungi occurred over the past decade with a total of over 1000 new metabolites having been described by the middle of 2010 (Rateb & Ebel 2011). Spanning 2005 to mid-2010, over 600 new metabolites were described, originating from over 200 fungal strains isolated predominantly from algae (21%), sponges (19%), sediments (16%), and mangroves (15%) (Rateb & Ebel 2011). However, only five of these isolates represented marine fungi (two of which were isolated and previously studied prior to 2002), and study of these organisms yielded only 19 new compounds. Clearly, the extensive resources dedicated to chemical investigations of marine fungi have contributed relatively little to the understanding of the chemistry of marine fungi (*sensu strictu*).

## New metabolites described from marine fungi (*sensu strictu*)

### Ascomycetes

*Aigialus parvus* is a Pleosporalean marine ascomycete that has been repeatedly isolated from submerged roots and branches of several mangrove plants as well as from submerged wood panels used as bait for the isolation of marine fungi (Kohlmeyer & Schatz 1985; Alias et al. 1995; Tan et al. 1995). From raised stromatic, ostiolate fruitifications, this fungus extrudes ascospores having apical and subapical cells covered by a gelatinous sheath or cap, to aid in the attachment of spores to substrata (Kohlmeyer & Schatz 1985). A strain of *A. parvus* (BCC 5311) was isolated from mangrove wood and metabolite production of this strain in culture was extensively studied. In 2002, authors reported the isolation of 14-membered resorcylic acid lactones, the known metabolite hypothemycin and novel aigialomycins A-E (**1–5**), of which both hypothemycin and aigialomycin D demonstrated antiplasmodial activity (Isaka et al. 2002) and cytotoxicity against human cell lines (where cytotoxicity was attributed to CDK/GSK-3 inhibition) (Barluenga et al. 2006). Due to the slow-growing nature of the fungus in culture, the strain was grown for 80 days, after which the new spiroketal metabolite aigialospirol (**6**) and a different class of metabolite having a ketene acetal functionality, aigialone (**7**), were isolated (Vongvilai et al. 2004). Follow-up investigations led to the discovery of additional aigialomycins F (**8**) and G (**9**), 7′,8′-dihydroaigialospirol (**10**) and 4′-deoxy-7′,8′-dihydroaigialospirol (**11**) (Isaka et al. 2009).

**Figure:**
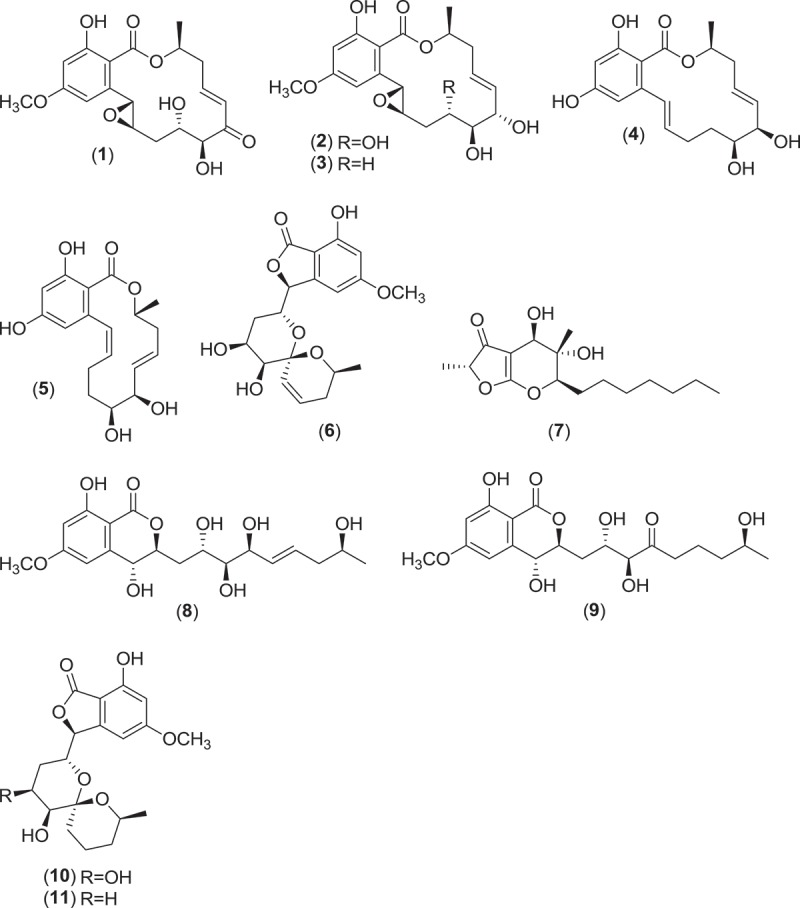

Another Pleosporalean marine genus, *Helicascus*, is comprised of two taxa that produce a characteristic coiled endoascus that uncoils upon spore release (Jones et al. 2009). The type species *Helicascus kanaloanus* (ATCC 18591) was first isolated from wood of dead proproots and young *Rhizophora mangle* plants in a Hawaiian mangrove swamp (Kohlmeyer 1969). Ejected ascospores were found to accumulate around the pores of stromatic fruiting bodies found *in situ* that were slightly raised above the substratum surface. These spores were germinated to isolate the fungus in culture. Following deposition in a public culture collection, the isolate was studied for the production of secondary metabolites. Two new diastereomeric δ-lactones, helicascolides A (**12**) and B (**13**), were isolated along with the previously known metabolite (*S*)-(+)-ochracin as the major components of the culture extract (Poch & Gloer 1989a).

**Figure:**
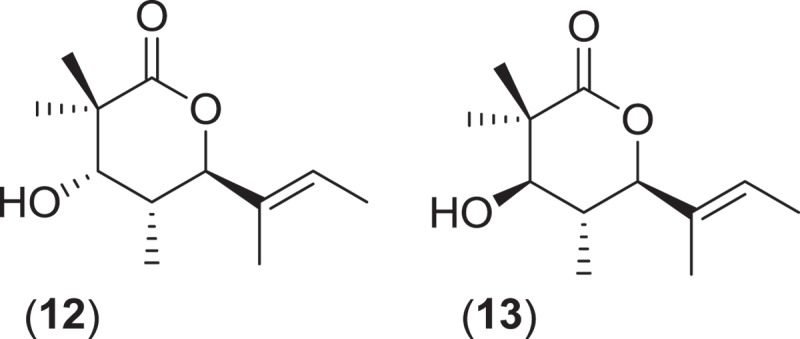

Within the Pleosporales, at least 13 species classified within the genus *Leptosphaeria* have consistently been reported from marine environments occurring as saproprobes on intertidal wood and plant material in mangroves and salt marshes (Gessner 1977; Kohlmeyer & Kohlmeyer 1979). These species produce ostiolate ascomata, usually without an associated stroma, that are found immersed within the substratum (Jones et al. 2009). From systematic studies, the genus *Leptosphaeria* was found to be polyphyletic, and several of the marine taxa previously residing in *Leptosphaeria* have been reassigned to the genus *Phaeosphaeria* (Khashnobish & Shearer 1996; Camara et al. 2002; Suetrong et al. 2009). Investigations into secondary metabolite production from different strains of the marine fungus *Phaeosphaeria orae-maris* (=*Leptospaeria orae-maris*) originating from different cellulosic substrata led to the isolation of several structurally similar compounds; leptosphaerin (**14**), leptosphaerolide (**15**), and obioninene (**16**) (Schiehser et al. 1986; Miller & Savard 1989; Guerriero et al. 1991). Discovery work involving another marine fungus, *P. spartinae* (=*L. spartinae*) isolated from the inner tissues of the marine alga *Ceramium* sp., resulted in the characterization of a number of new metabolites. Spartinoxide (**17**), the enantiomer of the known compound A82775C, spartinols A-D (**18–21**), and the bicyclic-spartinols furanospartinol (**22**) and pyranospartinol (**23**) are new polyketides obtained from *P. spartinae* (Elsebai 2011), with spartinoxide and spartinol C demonstrating inhibition of the enzyme human leukocyte elastase, a disease target associated with pulmonary emphysema, rheumatoid arthritis, and cystic fibrosis (Elsebai et al. 2009, 2010). Additionally, the strain produced the known metabolites 4-hydroxy-3-prenyl-benzoate and anofinate (Elsebai et al. 2009) along with an unusual metabolite (spartopregnenolone; **24**), that bears a structural resemblance to the triterpenoid lanosterol as well as the steroid pregnenolone (Elsebai et al. 2013).

**Figure:**
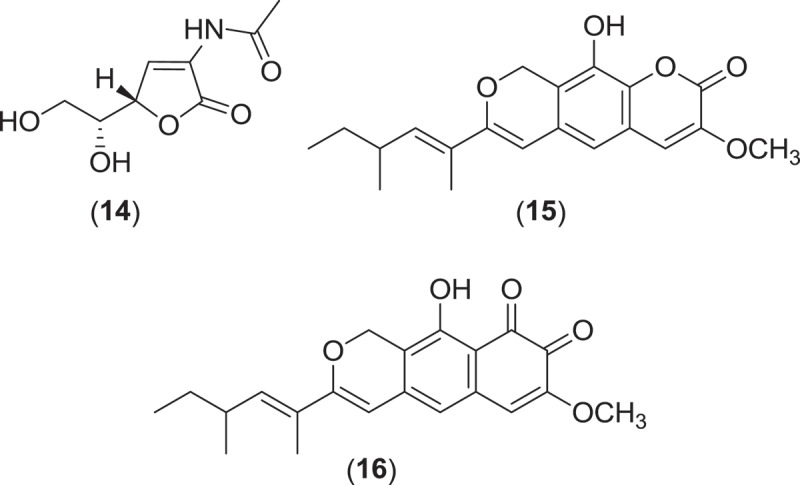

**Figure:**
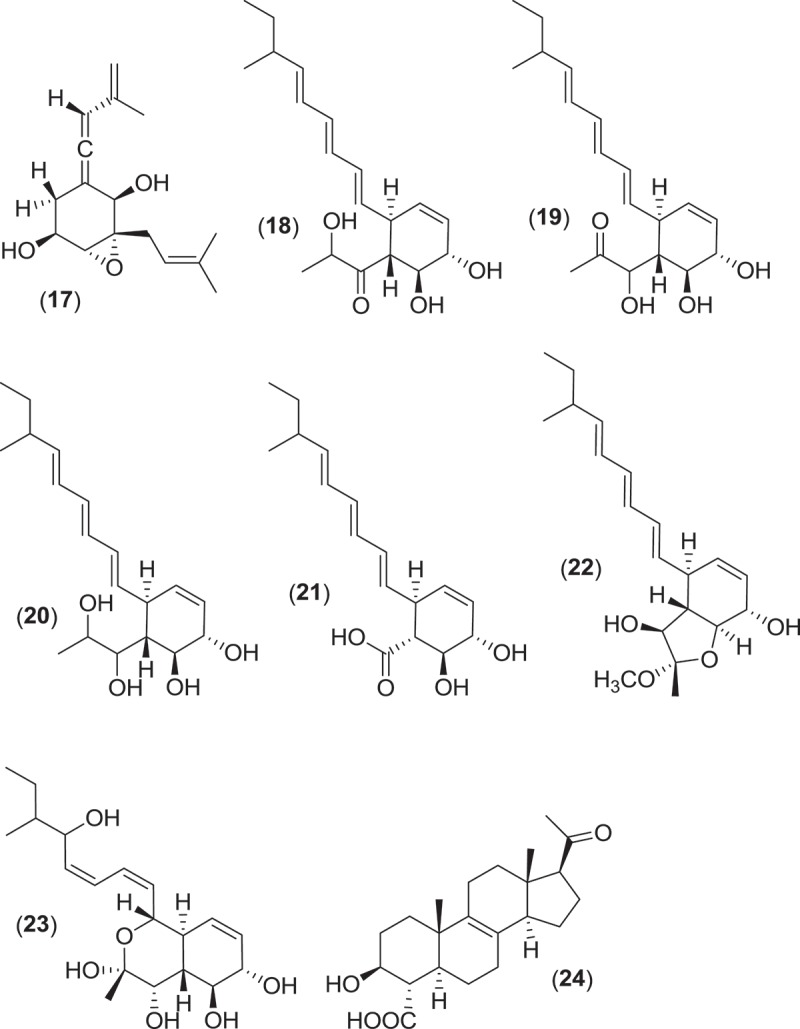

**Figure:**
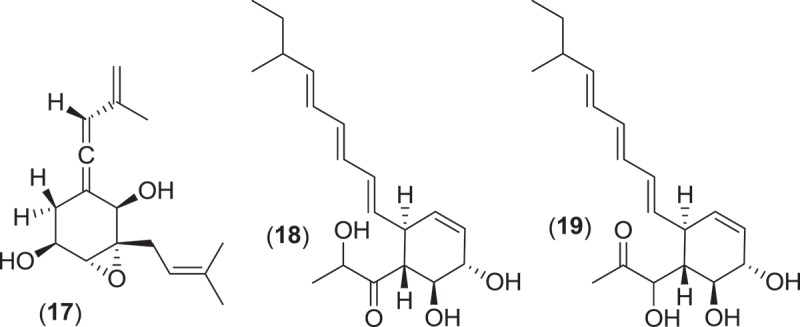

*Byssothecium obiones* (Pleosporales), a common species occurring on senescent culms of the estuarine marsh grass *Spartina alterniflora*, has previously been assigned to several different genera including *Leptosphaeria* (as *L. obiones*); however based on ITS sequence data, *B. obiones* was not found to belong to either *Leptosphaeria* or *Phaeosphaeria* (Khashnobish & Shearer 1996), rather it was assigned to *Byssothecium* in the *Teichosporaceae* due to the production of vericolourous ascospores (Barr 2002). The ostiolate, subglobose to ellipsoidal ascomata of *B. obiones* can be found immersed within the epidermis of senescent culms, often erumpent, producing 3-septate ascospores with hyaline end cells and brown central cells (Jones et al. 2009). Investigation of an isolate of the marine fungus *B. obiones* (identified under the name *L. obiones*), obtained from culms of *S. alterniflora*, led to the isolation of obionin A (**25**), a competitive inhibitor of the dopamine receptor D1 (Poch & Gloer 1989b) that is responsible for the regulation of signaling events in the central nervous system.

**Figure:**
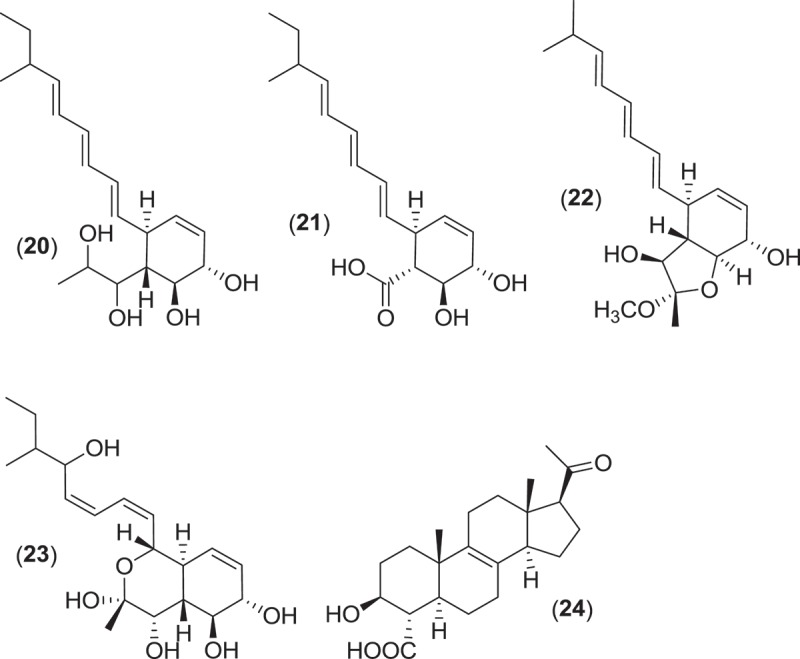

*Verruculina enalia* is another example of a Pleosporalean marine fungus that has been studied for the production of secondary metabolites. The genus *Verruculina* includes two species, with the obligate marine fungus, *V. enalia*, as the species ‘type’. Commonly isolated from driftwood in tropical mangroves, individual ostiolate, clypeate, black ascomata of *V. enalia* occur either partly or completely immersed within the substrata (Kohlmeyer & Volkmann-Kohlmeyer 1990). Short pedunculate asci can be found within a gelatinous matrix, containing ellipsoidal, monoseptate, dark brown, verrucose to verruculose ascospores that lack either a sheath or appendage (Kohlmeyer & Volkmann-Kohlmeyer 1990). An isolate of *V. enalia* collected from decayed wood of a *Casuarina* tree from a salt lake in the Bahamas was found to produce the new metabolites enalin A and B along with three previously described cyclodipeptides and a hydroxymethyl furfural metabolite (Lin et al. 2002). Although enalin A (**26**) and B (**27**) were not tested for biological activity, enalin A is a coumaranone, a metabolite class that is widely distributed in microorganisms and commonly associated with a number of antimicrobial activities (Lin et al. 2002).

**Figure:**
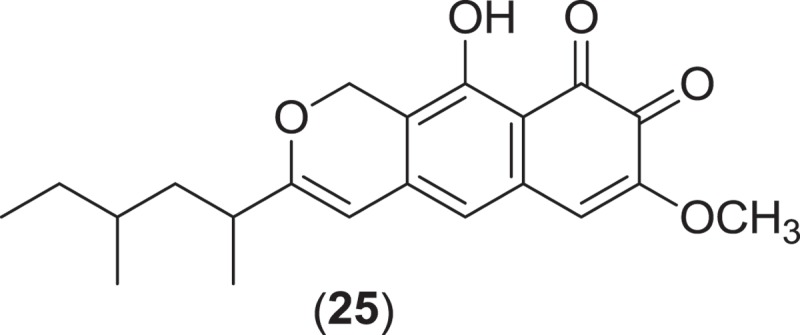

*Halokirschsteiniothelia maritima* (=*Kirschsteiniothelia maritima*) is a marine fungus from the order Mythilinidales (Boonmee et al. 2012) that has been studied for the production of secondary metabolites. Occurring on driftwood and drifting bark, intertidal and submerged wood, and test panels, *H. maritima* is primarily isolated as a saprophyte from coniferous wood from temperate marine habitats (Kohlmeyer & Kohlmeyer 1979). Superficial and gregarious ascomata from this species are observed as semiglobose, ostiolate, short papillate, and carbonaceous, containing brown, monoseptate ascospores with acute ends (Jones et al. 2009). An isolate of *H. maritima* obtained from submerged wood was found to produce the aromatic aldehyde metabolite ascochital (**28**), which inhibited the growth of *Bacillus subtilis* (Kusnick et al. 2002).

**Figure:**
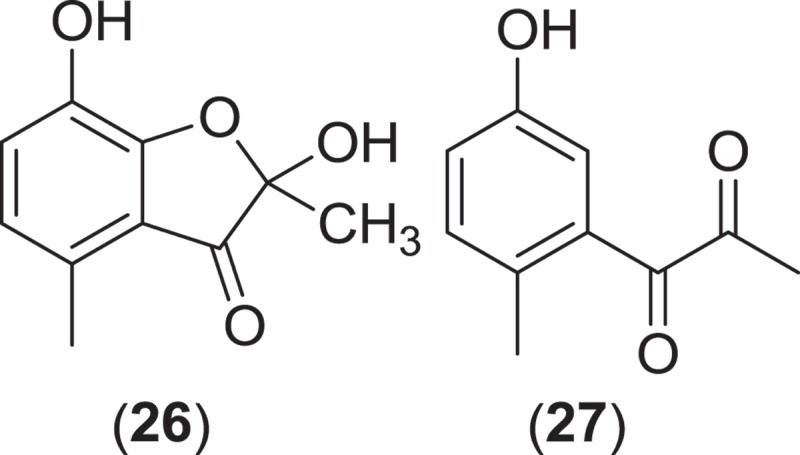

From the order Xylariales, *Halorosellinia oceanica* (syn. *Hypoxylon oceanicum*) is a marine ascomycete fungus that is associated with mangrove habitats. *Halorosellinia oceanica* produces dark, soft to leathery pseudostroma on decorticated wood and is associated with a *Nodulisporium* anamorph (Jones et al. 2009). This fungus was first studied for secondary metabolite production by Wyeth (Abbanat et al. 1998), which led to the discovery of a number of lipodepsipeptides (15G256ε, 15G256γ, 15G256δ (**29–31**)) and macrocyclic polyethers (15G256α, 15G256β, 15G256α-1, 15G256ι, and 15G256ω (**32–36**)), all of which demonstrated antifungal activity along with the linear polyesters 15G256α-2, 15G256β-2, 15G256ο, 15G256ν, and 15G256π (**37–41**), which did not inhibit fungal cell wall biosynthesis (Schlingmann et al. 1998, 2002). Additionally, further research into another strain of *H. oceanica* yielded the ophiobolane sesterterpenes halorosellinic acid (**42**), which demonstrated antiplasmodial activity, and 17-dehydroxyhalorosellinic acid (**43**), the isocoumarin glucosides halorosellins A and B (**44,45**), 2-hexylidene-3-methylsuccinate- 4-methyl ester (**46**), 4,8-dihydroxy-6-methoxy-4,5-dimethyl-3-methyleneisochroman-1-one (**47**), and 3-acetyl-7-hydroxy-5-methoxy-3,4-dimethyl-3*H*-isobenzofuran-1-one (**48**), along with the previously described known metabolites 2-hexyl-idene-3-methylsuccinate, cytochalasin Q, and 5-carboxymellein (Chinworrungsee et al. 2001, 2002). Compound SZ-685C from *Halorosellinia* sp. 1403C (**49**) is an emodin-like anthraquinone and an inhibitor of protein kinase B. The compound was active both *in vitro* in various cancer cell line assays and *in vivo* in a breast cancer xenograft mouse model where it induced apoptosis and suppressed the Akt/FOXO pathway at low mM concentrations (Xie et al. 2010; Chen et al. 2013; Wang et al. 2013).

**Figure:**
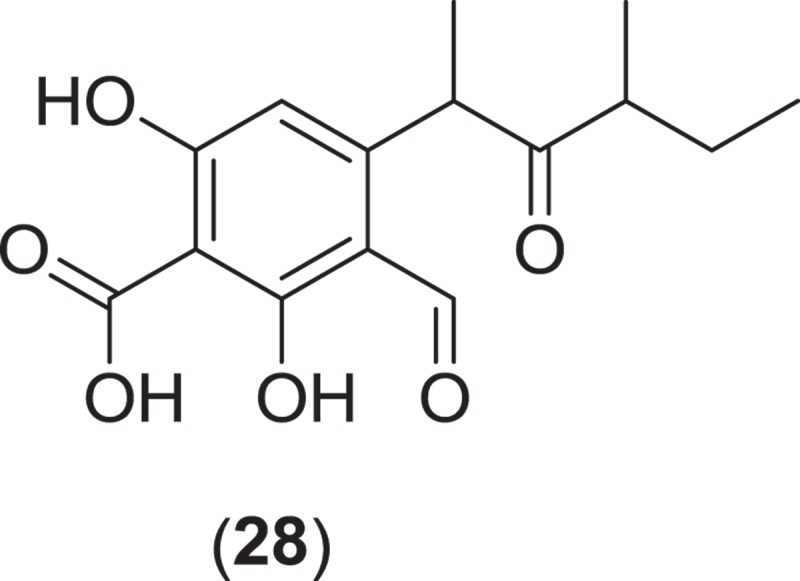

**Figure:**
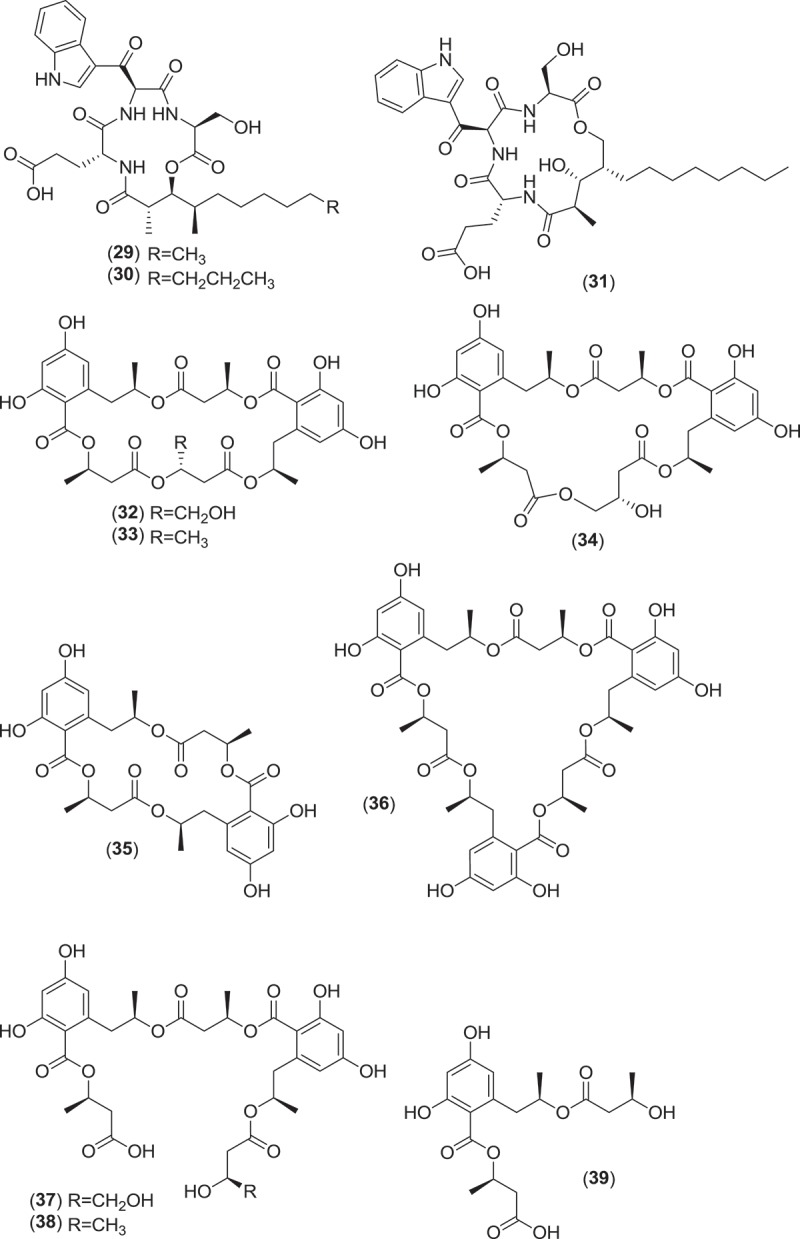

*Kallichroma* is a genus of Hypocrealean marine fungi composed of two species, *K. glabrum* and *K. tethys*. Species of this genus are saprobic and have primarily been isolated from mangrove wood in subtropical to tropical habitats (Kohlmeyer & Kohlmeyer 1979). Both species form solitary or gregarious, ostiolate orange–brown to orange–yellowish ascomata that occur immersed within the substratum and sometimes erumpent bearing asci that deliquesce to release ellipsoidal to ovoid, monoseptate ascospores that lack appendages or a sheath (Jones et al. 2009). An isolate of *K. tethys* was found to produce a new culmorin analog, the tricyclic sesquiterpene isoculmorin (**50**) (Alam et al. 1996). Although the authors indicated that further studies of the strain were in progress, no further work involving the secondary metabolism of *K. tethys* has been published.

**Figure:**
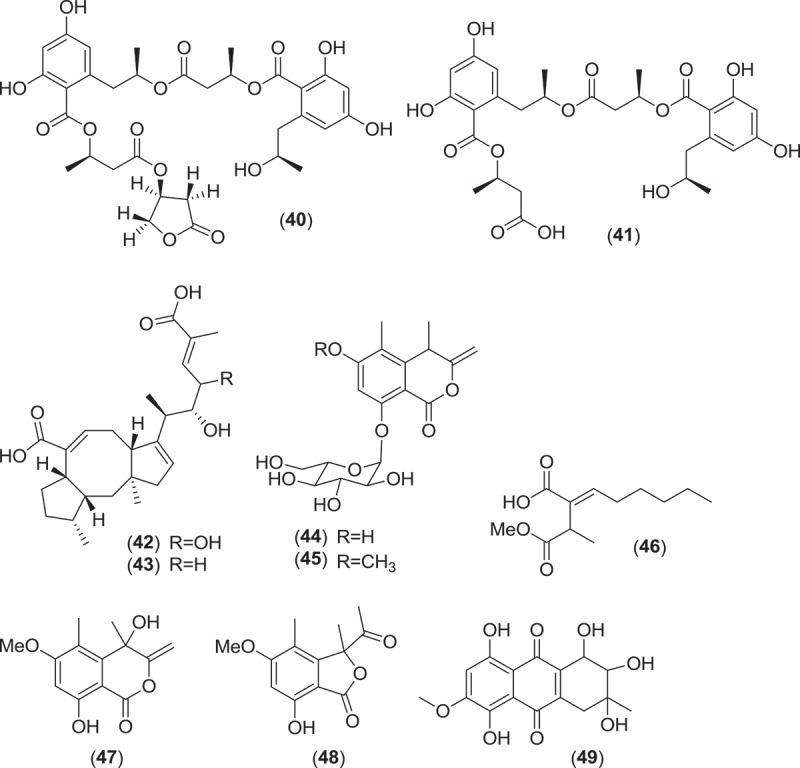

The genus *Corollospora* is composed of arenicolous marine fungi, classified within the order Halosphaeriales, and having anamorphs from three different genera (Jones et al. 2009). Ascomata of this genus typically lack ostioles, produce ascospores with appendages, form in culture in shake flasks with sand grains or other solid substrata, and are readily isolated from sea foam, marine sand, and driftwood (Kohlmeyer & Kohlmeyer 1979; Jones et al. 1983; Ramakrishna & Sabaratnam 2002). Two different *Corollospora* spp. have previously been studied for secondary metabolite production. An isolate of *C. maritima* was obtained from driftwood, and culture extracts yielded the new phthalide metabolite, corollosporine (**51**), which demonstrated antibacterial activity against *Staphylococcus aureus, Bacillus subtilis*, and *Escherichia coli* (Liberra et al. 1998). The second species studied was *C. pulchella*, also isolated from driftwood. The metabolite pulchellalactam (**52**) was isolated from this strain and was of particular interest as it exhibited inhibitory activity in a cell-free assay against the receptor-like transmembrane protein tryrosine phosphatase CD45 (Alvi et al. 1998). The previously described antimicrobial dioxopiperazines melinacidins II-IV and gancidin W have also been observed as metabolites of a *C. pulchella* isolate obtained from sand grains (Furuya et al. 1985). Phylogenetic studies of the *Halosphaeriales* include the anamorphic genus *Varicosporina* within the genus *Corollospora* (Campbell et al. 2002)*. Varicosporina* is composed of three anamorph taxa (Jones et al. 2009) commonly found from plant and algal detritus and sea foam from both tropical and subtropical waters (Kohlmeyer & Kohlmeyer 1979). *Varicosporina ramulosa* produces acrogenous, septate, irregularly staurosporous conidia from simple, geniculate conidiophores (Meyers & Kohlmeyer 1965). Several macrodiolides were isolated from a culture extract of a *V. ramulosa* strain isolated from a sample of an alga (*Cytoseira* sp.), including the known metabolites grahamimycins A1, colletodiol, and colletoketol along with the new dihydrocolletodiol analogs 9,10-dihydro-(6*R*,11*S*,12*S*,14*R*)-colletodiol (**53**) and 9,10-dihydro-(6*R*,11*R*,12 *R*,14*R*)-colletodiol (**54**) (Höller et al. 1999).

**Figure:**
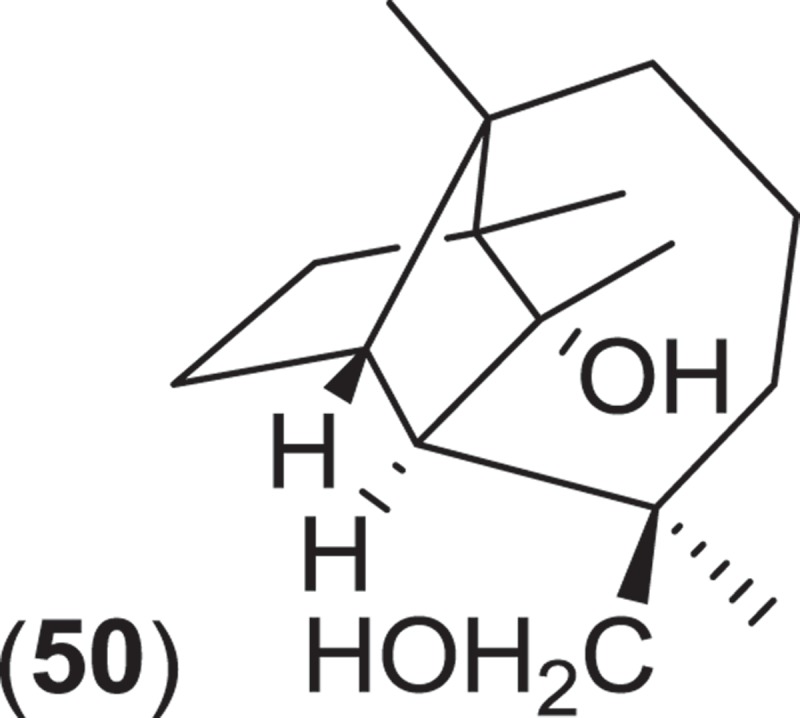

**Figure:**
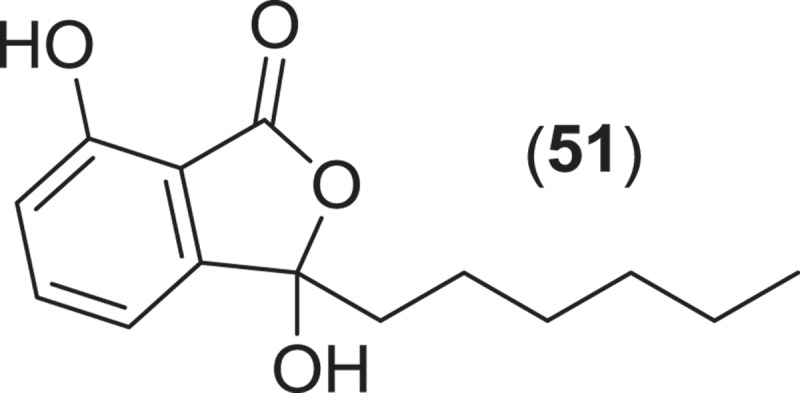

**Figure:**
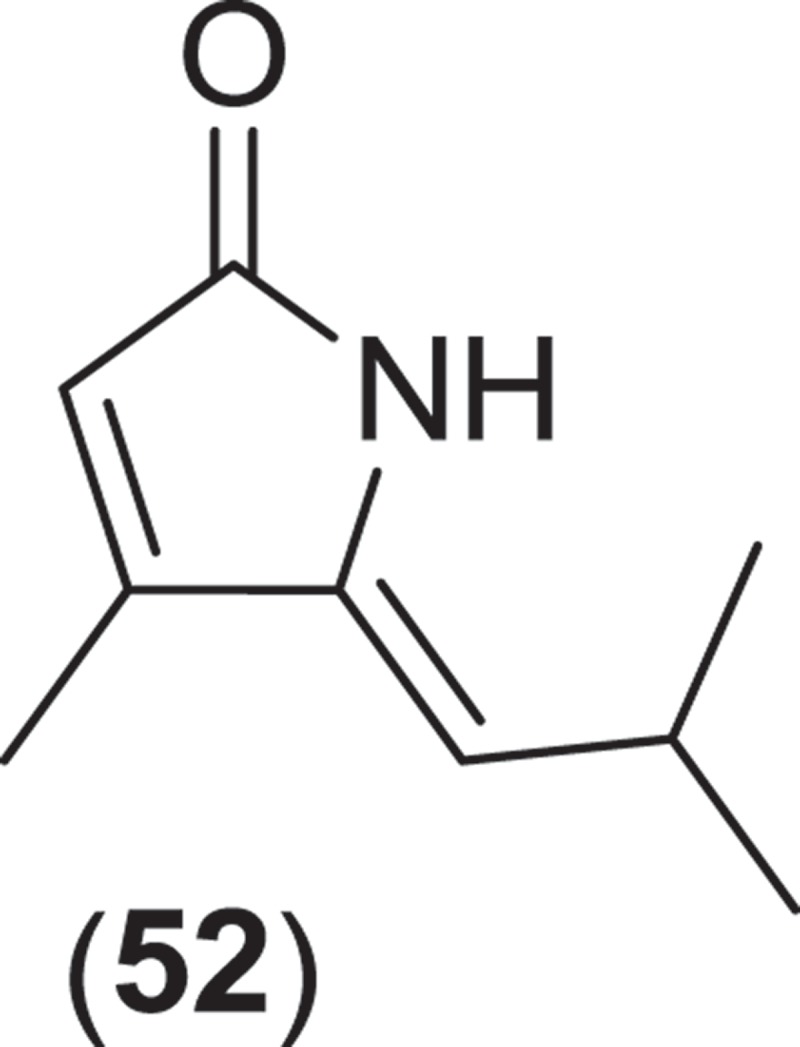

*Lignincola* is an example of another cosmopolitan genus of obligate marine fungi of the *Halosphaeriaceae*, composed of four taxa (although taxonomic placement of two of the species within the genus remains tentative), that occur on submerged and intertidal woody substrata in both temperate and tropical waters (Kohlmeyer 1984; Jones et al. 2009; Liu et al. 2011). Fructifications on substrata occur as immersed to almost superficial, ostiolate ascomata that extrude single septate ascospores that lack appendages (Kohlmeyer 1984). Investigation into the secondary metabolism of an isolate of *L. laevis* yielded an unusual dimerized phosphorohydrazide thioate (**55**) that exhibited potent cytotoxicity against a mouse murine leukemia cell line (Abraham et al. 1994).

**Figure:**
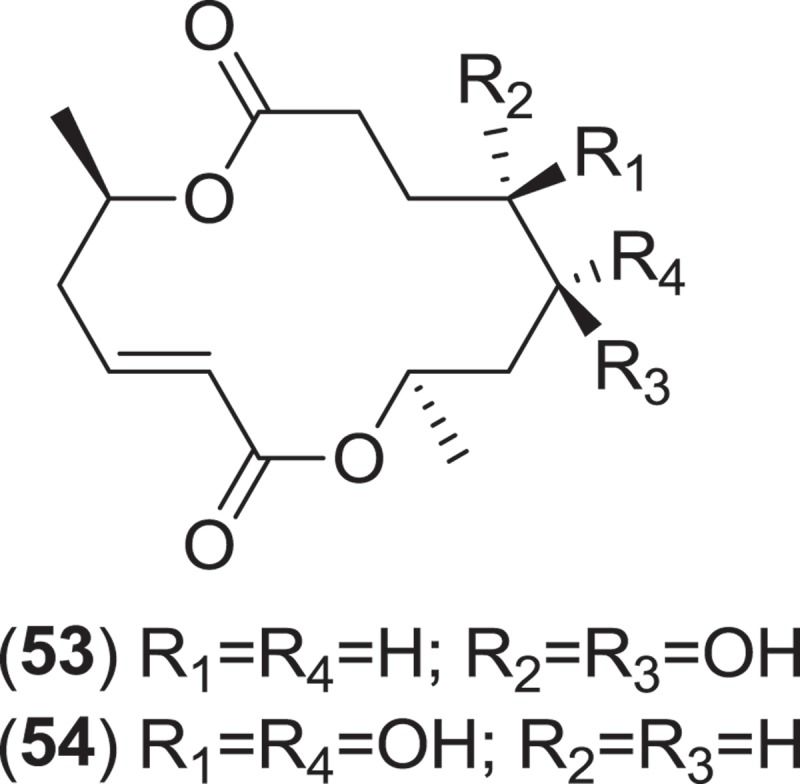

From the Sordariales, two species from the genus *Zopfiella* have been investigated for metabolite production. *Zopfiella* spp., when found fruiting on marine substrata, produce solitary, superficially immersed, globose to subglobose ascomata that are decorated with septate branched hairs and asci that deliquesce to release ellipsoidal, 1-septate ascospores that can readily be identified as they are composed of a large apical olivaceous cell and a smaller hyaline basal cell which lack appendages or a sheath (Jones et al. 2009). Both species produce a *Humicola*-like anamorph. A strain of the obligate marine species, *Z. marina*, isolated from marine mud (obtained from a depth of 200 m) was found to produce zopfinol (**56**), a chlorinated phenol with an aliphatic side chain, and the potent antifungal metabolite zofimarin (**57**), a sordarin derivative (Kondo et al. 1987; Ogita et al. 1987). *Zopfiella latipes* is a facultative marine fungus that has been reported from various sea grasses as well as both drift and submerged wood (Jones et al. 2009). Two new pyrrolidinone derivatives, zopfiellamides A (**58**) and B (**59**), were obtained from a strain of *Z. latipes* isolated from marine sediment from the Indian Ocean (Daferner et al. 2002). Both of the zopfiellamides demonstrated moderate antimicrobial activity against Gram-positive and Gram-negative bacteria as well as two yeast strains (Daferner et al. 2002).

**Figure:**
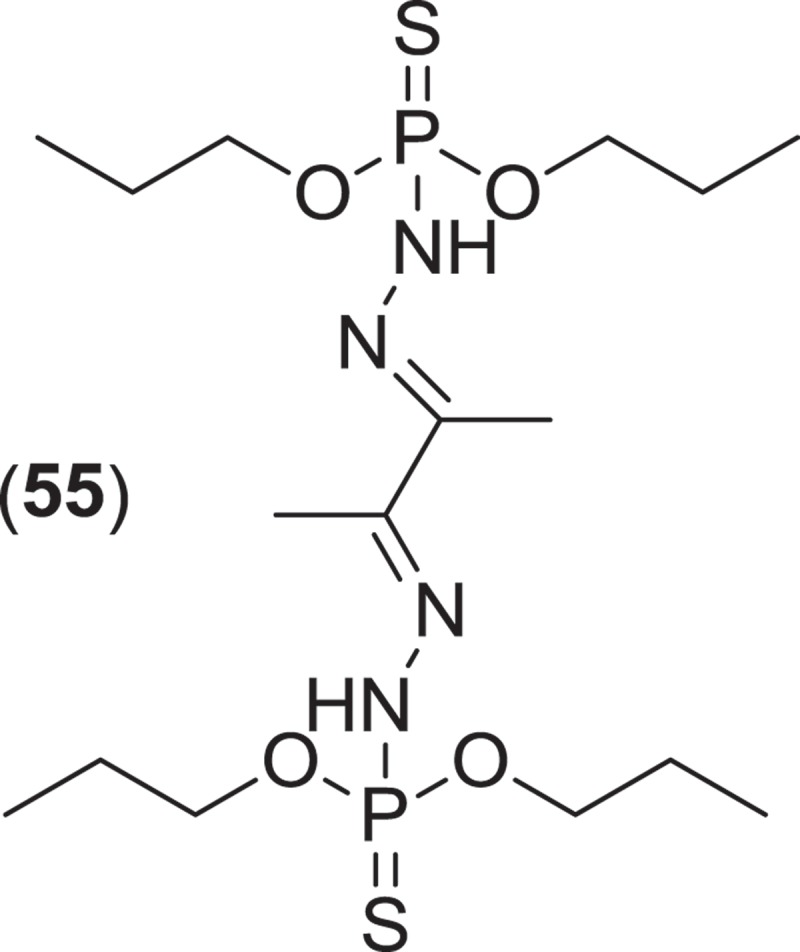

**Figure:**
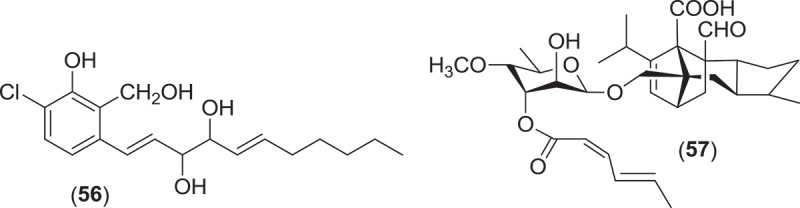

## Anamorphic ascomycetes

Several anamorphic marine fungi (*Ascochyta salicorniae, Asteromyces cruciatus*, and *Paradendryphiella salina*) have been investigated for secondary metabolite production. Each of these species is saprobic on algae and other cellulosic substrata and is commonly isolated from marine detritus, marine sand, and sea foam (Kohlmeyer & Kohlmeyer 1979). *Asteromyces cruciatus* is characterized by hyaline to brown hyphae from which arise cylindrical to subclavate conidiogenous cells bearing lateral whorls of up to 12–14 ovoid conidia on denticles (Kohlmeyer & Kohlmeyer 1979; Jones et al. 2009). Investigations into the secondary metabolite production of several strains of *A. cruciatus* led to the isolation of the new metabolite 2,4-dimethyl-4,5-dihydrofuran-3-carbaldehyde (**60**), and the previously described metabolites 3,5-dimethyldihydrofuran-2-one (Höller et al. 1999), regiolone (Gulder et al. 2012), and the epipolythiopiperazinedione antibiotics gliovictin, ^1^ *N*-norgliovictin, bis-*N*-norgliovictin, and hyalodendrin (Shin & Fenical 1987; Gulder et al. 2012). A recent investigation of a strain of *A. cruciatus* (obtained from algal detritus) was grown in a battery of different media conditions designed to diversify the production of low-abundance metabolites. One of these fermentations yielded a new cyclic pentapeptide, lajollamide A (**61**) (Gulder et al. 2012). In our opinion, this fungus was the only marine fungus (*sensu strictu*) that appeared in a special issue of the journal *Marine Drugs* (2011), entitled ‘Bioactive Compounds from Marine Fungi’, however, there were a substantial number of fungi mentioned in that issue that were reported as unidentified.

**Figure:**
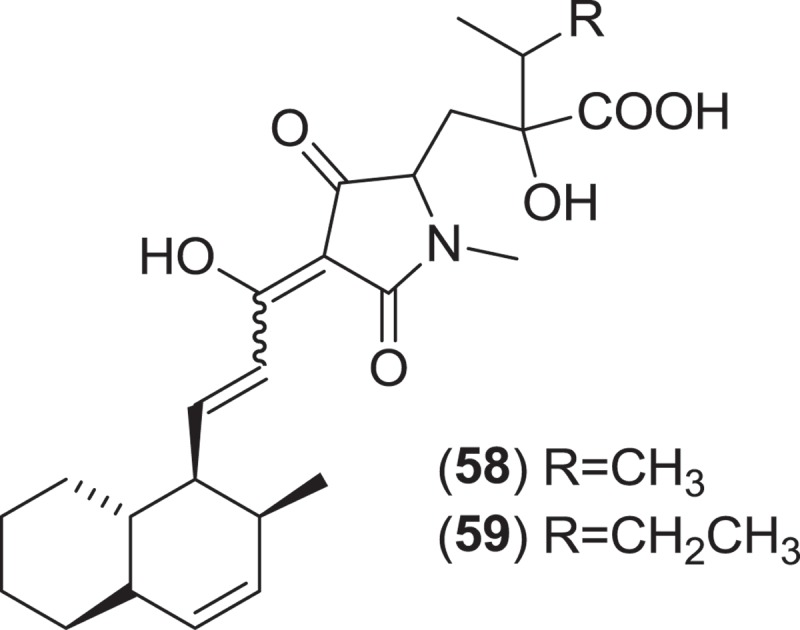

*Paradendryphiella arenaria* and *P. salina* are two marine species that comprise the anamorph genus *Paradendryphiella*. Originally included in the anamorph genus *Dendryphiella*, the genus *Paradendryphiella* was recently erected following a multilocus phylogenetic analysis that classified both marine species as a unique taxon within the *Pleosporaceae* and distinct from the type species of *Dendryphiella* (Woudenberg et al. 2013). These fungi readily sporulate on various marine substrata and produce ellipsoidal to cylindrical, 1–11 septate, pale brown to olivaceous conidia bearing short, non-denticulate stalks (Kohlmeyer & Kohlmeyer 1979; Jones et al. 2009). Conidiophores are characterized as being simple, macronematous, apically swollen and pale brown to olive brown, having conspicuous scarring once conidia have been shed (Kohlmeyer & Kohlmeyer 1979; Jones et al. 2009). Investigations into the secondary metabolism of a *P. salina* strain yielded a diverse group of new trinor-eremophilanes and eremophilanes. Dendryphiellin A (**62**) was the first of the trinor-eremophilanes to be isolated and characterized (Guerriero et al. 1988), followed by the discovery of dendryphiellins B-D (**63–65**) and the eremophilanes, dendryphiellins E (**66**) and G (**67**) (dendryphiellin F was an ethanolysis artifact from the extraction process) (Guerriero et al. 1989). Additional dendryphiellins A1 (**68**), E1 (**69**), and E2 (**70**), and the glyceryl ester of the dendryphiellin side chain, glyceryl dendryphiellate A (**71**) were subsequently isolated from the same *P. salina* strain (Guerriero et al. 1990).

**Figure:**
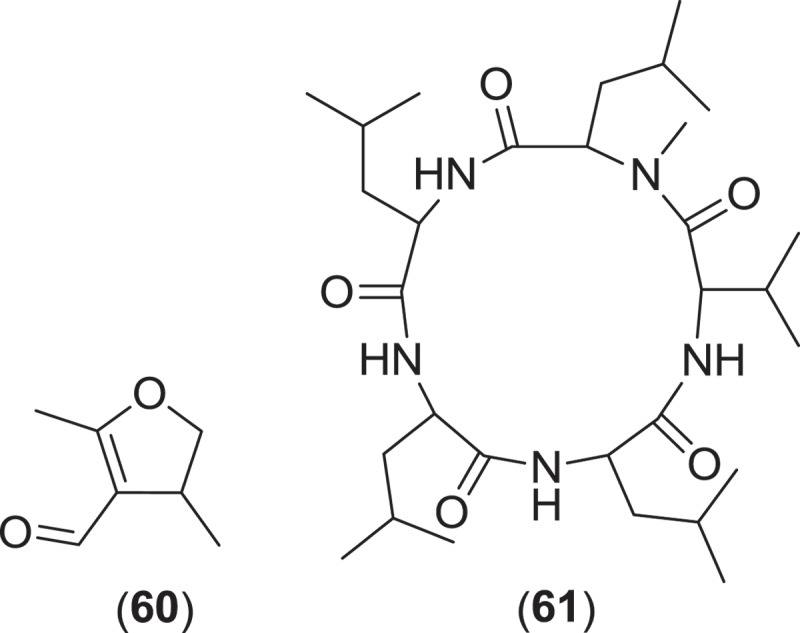

*Ascochyta salicorniae* is a saprobic, sometimes parasitic, marine coelomycete that usually sporulates on halophytes (salt marsh plants and algae) after plants senesce and desiccate (Kohlmeyer 1981; Jones et al. 2009). Pycnidia erupt from under the epidermis of the lower, dry branches of marsh plants, e.g., *Salicornia* spp., eventually spreading over the desiccated plant (Kohlmeyer 1981). The ostiolar canal of *A. salicorniae* and other similar saprobic marine coelomycetes are lined with gelatinous material or mucilage which probably prevents penetration of water into the canal (Kohlmeyer 1981). A strain of *A. salicorniae*, isolated from a green alga (*Ulva* sp.) from the North Sea, was the focus of investigations into secondary metabolite production from this species. The first new metabolites discovered were ascosalipyrrolidinones A (**72**) and B (**73**) and ascosalipyrone (**74**), where **72** was found to inhibit the protein target tyrosine kinase p56, had antiplasmodial activity against *Plasmodium falciparum* (strains K1 and NF 54), and inhibited growth of *Bacillus megaterium, Microbotryum violacea*, and *Mycotypha microspora* (Osterhage et al. 2000). Further work on this fungus yielded two more epimeric compounds, ascolactones A (**75**) and B (**76**) (Seibert et al. 2006), along with two new spriroketal-containing metabolites, ascospiroketals A (**77**) and B (**78**) (Seibert et al. 2007). The known compounds ascochital (previously isolated from the marine fungus *K. maritima*), ascochitine, hyalopyrone, and 2,3-dihydro-2-hydroxy-2,4-dimethyl-5-*trans*-propenylfuran-3-one were also encountered (Osterhage et al. 2000; Seibert et al. 2006). Ascochitine was found to inhibit the enzymatic activity of MPtpB (*M. tuberculosis* protein tyrosine phosphatase 1B) (Seibert et al. 2006).

**Figure:**
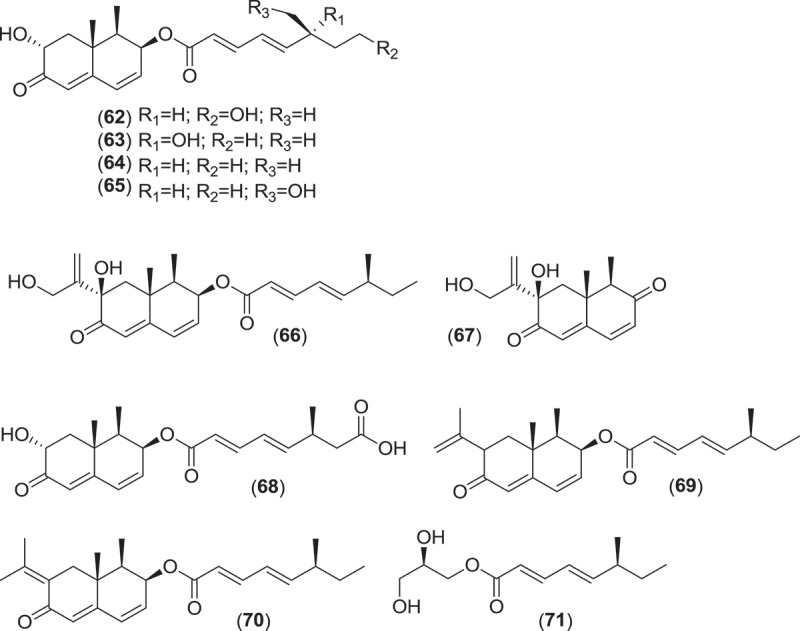

**Figure:**
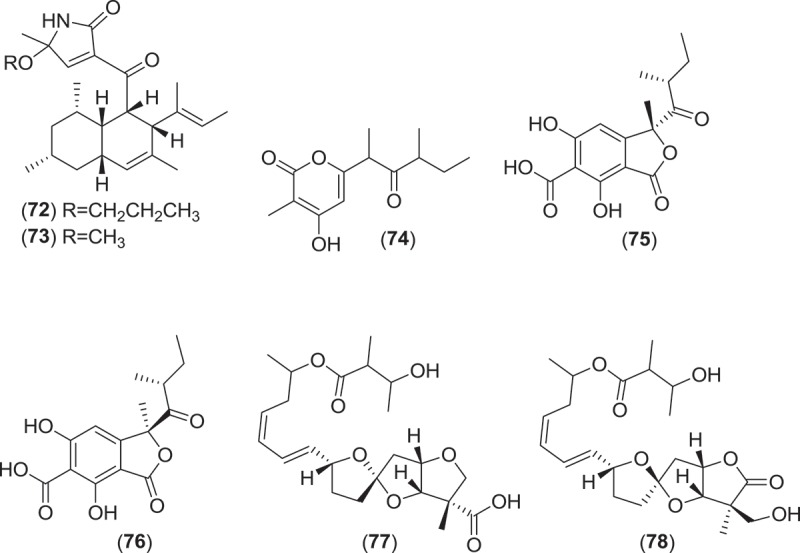

## How unique are the natural products produced by marine fungi (*sensu strictu*) compared with the diversity encountered from terrestrial fungi?

As illustrated above, the natural products reported from marine fungi (*sensus strictu*) are as diverse in chemical structure and biosynthetic origin as those produced by their terrestrial counterparts. Phylogenetic evidence indicates that the principal lineages of marine fungi (taxa of the Lulworthiales and Halosphaeriales along with certain species of the Capnodiales, Dothideales, Hypocreales, Hysteriales, Jahnulales, Mytilinidiales, Patellariales, Pleosporales, Sordariales, and Xylariales) were derived from terrestrial ascomycete lineages primarily from the Sordariomycetidae, Dothidiomycetidae, and Eurotiomycetidae (Campbell et al. 2005; Zhang et al. 2006; Schoch et al. 2007, 2009; Zuccaro et al. 2008; Suetrong et al. 2009). Sequencing of several hundred fungal genomes has now revealed that the main subclasses of the subphylum of filamentous ascomycetes (Pezizomycotina), namely, the Sordariomycetidae, Dothidiomycetidae, Leotiomycetidae, and Eurotiomycetidae, often have very rich secondary metabolomes, therefore it is logical to assume that the marine lineages derived from within these ascomycete subclasses would also have an active and complex secondary metabolism.

Assessment of whether or not marine fungi have evolved to produce secondary metabolites that are distinct from their terrestrial relatives is awaiting completion of genomic sequences. At the time of writing this review, the only genomes of marine fungi (*sensu strictu*) that have been sequenced are those of yeasts, including *Pseudozyma hubeiensis* (Konishi et al. 2013) and *Debaryomyces hansenii* (http://www.genolevures.org/deha.html). A genome of a filamentous marine ascomycete or basidiomycete that is likely to have biosynthetic gene clusters for secondary metabolites has yet to be released. That knowledge gap should be at least partially addressed soon because the 1000 Fungal Genome Project (Grigoriev et al. 2011) has targeted *Lindra thalassiae, Lulworthia fucicola, Torpedospora radiata*, and *Corollospora maritima* for genome sequencing. These genomes will enable identification of pools of candidate marine-adaptation genes. These data will lead to hypotheses about how isolation in the marine habitat may have influenced the evolution of secondary metabolite gene clusters in terms of divergence from ancestral pathways or loss or expansion of pathways for individual structural classes. Comparative data will also allow assessment of whether marine-derived fungi have diverged significantly from terrestrial counterparts (Penn & Jensen 2012). It will be interesting to know to what degree archaic secondary metabolite gene clusters (e.g., ferrichrome- and fusigen-type siderophores, 6-methyl-salicylic acid and orsellinic acid-type polyketides, and melanin and spore-pigment polyketides) that are widespread in terrestrial fungi have been conserved in marine fungi.

## Why has the rate of discovery of natural products from marine-derived fungi increased exponentially over time yet the rate of discovery from marine fungi (*sensu strictu*) has decreased?

The majority of the natural products that have been isolated from marine fungi (predominantly in the years prior to 2000) resulted from collaborative research between natural products chemists and professional mycologists, or from using authentic strains acquired from public culture collections that were taxonomically confirmed and deposited by marine mycologists. Most of these discoveries occurred prior to the general acceptance of the marine environment as a ‘hot spot’ for microbial natural products discovery. Complicating the landscape in recent years has been a void in scientific leadership in the field of marine mycology following the retirement of the eminent and iconic figures E.B. Gareth Jones and Jan Kohlmeyer (Jones 2012). Lack of active participation and guidance from the marine mycology community in fungal chemistry projects has likely contributed to the observed increase in the rate of discovery of natural products from marine-derived fungi rather than from marine fungi (*sensu strictu*). Obviously, cross-fertilization of marine mycologists with natural products chemists is needed, and therefore, opportunities are waiting for both.

Active mycological input into the design of marine natural products chemistry projects has largely been absent, but highly successful models for structuring active cooperation can be found in other subdisciplines of marine natural products. Perhaps the most exemplary example has been the microbiologist–chemist partnership of Paul Jensen and William Fenical at the Scripps Institute of Oceanography (Mincer et al. 2002; Jensen & Fenical 2005; Penn & Jensen 2012; Ziemert et al. 2014). Many of the same questions regarding marine-derived fungi have had parallels in strains of Actinomycetes isolated from the marine environment. Previously, the concept of marine Actinomycetes was not widely accepted by the marine microbiology community. Literature suggested that the vast majority of marine-derived Actinomycetes were of terrestrial origin. In fact, this statement seems to be true because analysis of near and offshore sediments indicates that Actinomycete populations are dominated by strains previously reported from nonmarine sources (Prieto-Davó et al. 2008). However, with support over many years from the USA’s NSF and NIH, Jensen and Fenical have sustained investments in testing hypotheses surrounding marine Actinomycetes and their potential as sources of new secondary metabolites. They have guided the recognition of novel and sometimes cryptic lineages of marine Actinomycetes from among the noise of terrestrially derived strains and have developed methods useful in discriminating marine-adapted species from those of terrestrial origin. Despite the predominant background of terrestrial genotypes, the Scripps team has applied a combination of ecology, physiology, and phylogenetics to recognize multiple lineages of marine-adapted Actinomycetales and other bacteria. Their conclusions have been supported by more recent comparative genomic data. Any proposed work on hypothesized recent divergences of apparent terrestrial fungal species into the marine environment should study carefully the lessons learned from this impressive body of work.

Another interesting collaborative model stems for the work of Rob van Soest, a marine biologist and authority on the systematics of the Porifera at the Naturalis Biodiversity Center. He has coauthored about 250 research articles, of which about half are in the field of natural products chemistry and chemical ecology where he participated in natural products research or supported chemists with authoritative identifications of specimens worldwide. Adaptations of such working models are acutely needed to advance the chemical understanding of marine fungi (*sensu strictu*).

**Figure 3.**
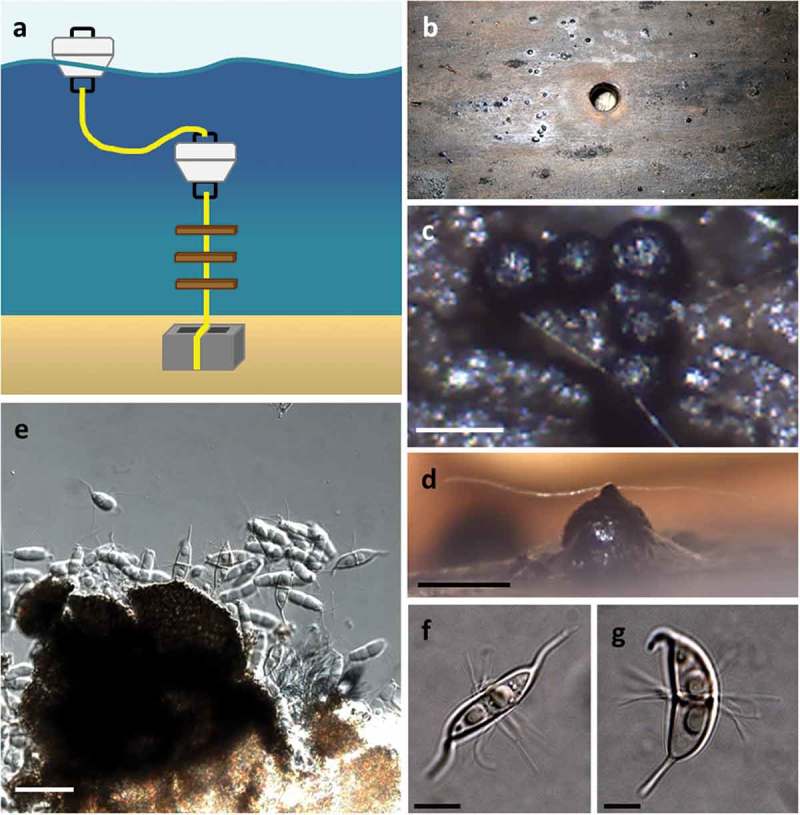
The use of baiting stations for the isolation of marine fungi. (a) Sterilized wooden boards are suspended in the water column. (b) Harvested wooden board after 6 months in damp chamber, ascomata are apparent on the surface. (c, d) Ascomata of *Corollospora maritima in situ* (scale bar = 250 µm). (e) Squash mount of *C. maritima* ascomata obtained from board (scale bar = 30 µm). (f, g) Ascospores of *C. maritima* with characteristic polar and equatorial appendages (scale bar = 10 µm).

Typical research funding structures and timelines in North America, Europe, and Asia are risk-adverse and work against sustained efforts to understand obligate marine fungi. A typical natural product chemistry project is funded on a two- to four-year cycle. If the project’s goal is based on isolation of natural products from marine fungi, the principal investigator will possibly employ one or more post-docs and graduate students. Without prior training, they lack the skills needed to painstakingly sift through drift wood, plant, and algal debris and to set up and examine baiting stations to capture marine fungi (Figure 3). Considerable experience and patience is required to recognize ascomata, basidomata, and conidiomata of marine fungi. The limited research schedule and pressure for results demand a high-throughput approach for obtaining isolates. Therefore, to avoid risk and for operational simplicity, the isolation techniques commonly reported in the literature by marine natural products isolation groups are dominated by plating methods where environmental samples are processed and plated out onto agar plates and incubated prior to the isolation of developing colonies. The methods employed in most of these studies do not differ significantly from those practiced more than 70 years ago (Sparrow 1937). These approaches favor the isolation of faster growing ubiquitous fungi, and although sea salts are included in the isolation medium, this approach selects for osmotolerant isolates, and thus contributes to the predominance of ubiquitous fungi that make up the largely predictable taxonomic composition of marine-derived fungi.

Granted, accumulation of significant numbers of strains of marine fungi (*sensu strictu*) can be a slow process. For example, in a recent 12-month survey of marine fungi on the shores of the Gulf of Mexico (Velez et al. 2013), a sampling of 600 debris samples yielded 21 taxa. An examination of 90 plant substrata samples collected on two Portuguese beaches yielded 35 species of fungi (Figueira & Barata 2007), while a more extensive sampling of 720 substrata samples along the length of Portugal’s coast yielded 56 marine species (Azevedo et al. 2012). Baiting of fungi with *Spartina maritima* stems in a Portuguese salt marsh yielded 26 species of marine ascomycetes and basidiomycetes (Barata 2006). Based on these examples, an academic team would need to work many years to accumulate enough obligate marine isolates to populate a screening library of effective size. However, the proven success of collection strategies such as baiting (using submerged wood panels and stakes in estuaries) or collections made directly from substrata (visual inspection in the field or from substrata incubated in damp chambers), to capture and isolate marine fungi argues for their inclusion in any natural product discovery project seeking metabolites from marine fungi.

**Figure 4.**
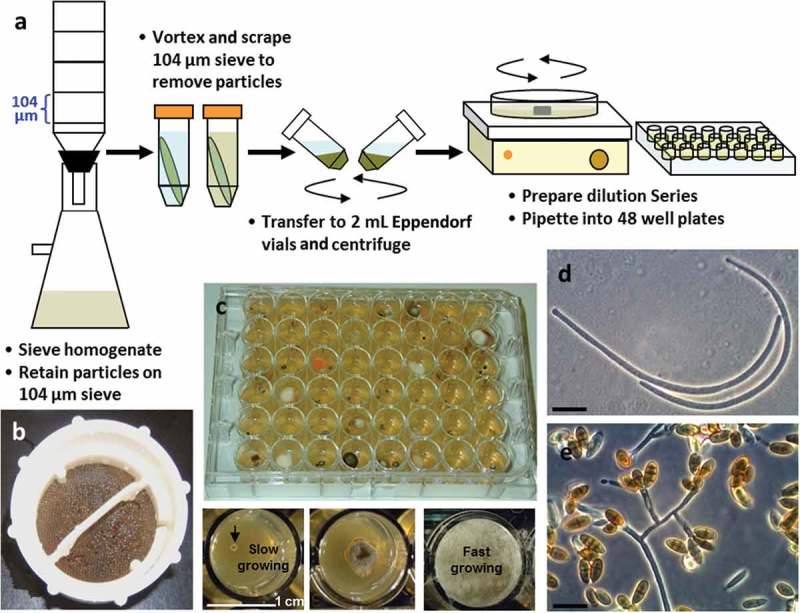
The use of particle filtration and dilution culturing in 48-well plates for the isolation of marine fungi. (a) Schematic of the particle filtration and dilution plating workflow. (b) Algal particles that were retained on filter sieve after homogenization and filtration. (c) Plating into 48-well plates allows for the separation of fast and slower growing isolates, increasing the isolation frequency of slower-growing fungi. (d) Conidia from a strain of marine fungus *Anguillospora marina* (telemorph = *Lindra obtusa*) isolated from algal tissue particles (scale bar = 20 µm). (e) Conidia and conidiophores of a strain of the marine fungus *Paradendryphiella arenaria* isolated from algal tissue particles (scale bar = 20 µm).

Alternative isolation strategies that would be able to capture vegetative cells of marine fungi embedded in their respective substrata could conceivably increase the rate of recovery of marine fungi. One technique that has proven effective in sampling from the marine environment and merits more widespread adoption is the combination of substrata particle filtration and dilution-to-extinction particle plating (Bills et al. 2004; see Figure 4 for an example). Cleaned substrata are deconstructed by homogenization and are filtered and washed through a series of meshes of decreasing pore sizes. Particles of a given size are then harvested and dispensed at high-dilution rates into 48- or 96-well plates (Collado et al. 2007; Unterseher & Schnittler 2009; Amend et al. 2010; Shrestha et al. 2011). The technique does not prevent the isolation of all spores, however, it does increase the rate of capture of fungi originating from vegetative fragments embedded in the matrix. Thus, slower-growing fungi (such as obligate marine fungi) actively growing within substrata particles can be separated and isolated from faster-growing taxa. Isolation of marine lineages can then be confirmed by sequencing rDNA marker genes or using PCR primers specific for known lineages. A recent search for fungi in ocean-submerged logs and driftwood in Arctic waters supports this idea and convincingly demonstrated the potential for effective high-throughput isolation of marine fungal lineages from such woody debris (Rämä et al. 2014). Fungi were isolated from 50 intertidal and sea-floor logs along the north coast of Norway, resulting in 577 strains classified into 147 operational taxonomic units by employing ITS sequences. Ascomycota dominated the isolates, but fungi of the Basidiomycota, Mucoromycotina, and Chytridiomycota were also isolated. Logically, because logs entered the marine environment pre-colonized by fungi, about half the strains were considered to have originated from the nonmarine environment. Nonetheless, significant numbers of strains with a high degree of taxonomic complexity from exclusive marine lineages of the Lulworthiales, Halosphaeriaceae, and other minor groups of obligate marine fungi were recovered. This study provides convincing evidence that deliberate long-term submerged incubation of sterilized wood or macro-algae, following by plating of micro-dissected wood or algal fragments or processing by dilution-to-extinction would yield pools of isolates highly enriched in marine fungi (*sensu strictu*).

Do the unidentified marine-derived strains currently present in the natural products literature represent new species of fungi? This question is difficult to answer because of several issues. In cases where mycological identifications have been outsourced, little to no information is provided in the publications regarding phenotypic characteristics used to determine species identity. Voucher strains are rarely deposited in public culture collections. Only in recent years have research groups started to use sequencing of bar-coding genes as a method of identification. A particular limitation, in many cases, is that, even when bar-coding genes have been used, the sequences have not been deposited in public databases, e.g., GenBank, or at a minimum, provided as text in the manuscript’s supplementary material. Furthermore, databases with reference sequences for marine fungi are still incomplete and impede the progress of molecular surveys of marine fungi (Rämä et al. 2014). Attempts to identify strains of marine taxa, such as Halosphaeriaceae (Microascales) and Lulworthiales, resulted in poor BLAST matches across the ITS region and uncertainty in the taxonomic assignments (Rämä et al. 2014). With the exponentially increasing rate of discovery of new natural products from fungi from marine substrata, the chances are high that some of these metabolites are originating from new fungal taxa not previously reported. However, until the same standard criteria required for publication in journals devoted to microbiology and fungal biology become routinely applied to the identification and characterization of isolates described in reports of new fungal chemistry, the accreditation of marine fungi as a true ‘hot spot’ of discovery cannot yet be proclaimed.

## Concluding remarks

Mycologists have compiled compendia of fungi and their substrata for decades and have left it up to the users to infer what the relationships were (Farr et al. 1989). Advertising of fungi isolated based solely on location, with the intention to imply a specialized function (e.g., Antarctic deep-sea-derived, marine-sediment-derived, alga-derived, sea hare-derived, fish-derived, mangrove-derived, insect-derived, lichen-derived, etc.), has become common practice. In our opinion, unless a clear relationship between the fungus and its substratum is evident, we believe that providing the best identification possible and noting the source of the isolate is sufficient; users will make their interpretations.

Ever since the initial efforts in this area were undertaken, the rate of discovery of new natural products from ‘marine-derived’ fungi has increased exponentially. However, this exponential rate of natural product discovery has not translated into a concurrent increase in knowledge of secondary metabolism from lineages of acknowledged marine fungi. The few reports of chemistry from marine fungi (*sensu strictu*) suggest that they will be an excellent source of new chemical entities, often associated with a variety of different biological activities.

The capacity for natural product biosynthesis is deep and expansive within the vast fungal kingdom, but few opportunities to explore truly virgin lineages of filamentous fungi for natural products remain, and obligate marine fungi comprise one of these still relatively virgin areas. In order to address the ultimate goal of discovering new bioactive natural products, the implementation of focused strategies aimed at the isolation of marine fungi (*sensu strictu*) is essential. In order to validate the isolation of ‘unknown’ fungi as being unique to science, genetic identification of strains using bar-coding genes (such as ITS rDNA sequences) and cataloging of these sequences in public repositories (e.g., GenBank) should be adopted. Access to these data will also aid in determining whether marine isolates are genetically distinct from those of terrestrial origin. Divergent adaptation is known to promote reproductive isolation in the Fungi, and such processes are likely widespread among fungi. Some of the prevalent types of marine-derived fungi, especially aspergilli and penicilia, are backed by an extensive phylogenetic, chemotaxonomic, and genomic infrastructure and, therefore, are approachable models to test evolutionary hypotheses about whether subpopulations of these fungi have migrated to and have become established within the marine environment. The pioneering work on marine Actinomycetes from the Scripps Institute of Oceanography can serve as a guide for how to approach these questions.

The majority of the natural products that have been isolated from marine fungi (*sensu strictu*) have resulted from collaborations between natural products chemists and mycologists. Continued success will necessitate active cooperation between natural product chemists and mycologists interested in the chemistry of marine fungi. Interests in chemical applications in marine biology could in turn attract a much-needed research investment in the field of marine mycology. Continued isolation of marine-derived fungi on nonselective media will likely continue the stream of research on ubiquitous fungi, many of which have already been extensively studied for natural products. Those seeking new natural products from marine fungi (*sensu strictu*) need to understand that obtaining new strains alone maybe insufficient. Working with these fungi is risky; many of these organisms will be difficult to grow, and conventional shake flask techniques alone will likely be inadequate to express their full spectrum of metabolites. In the near future, genome sequencing of targeted fungi will likely allow for a comprehensive assessment of their secondary metabolic potential and suggest strategies for gene expression, thus enhancing opportunities for finding new chemistry from these fungi.

As is the goal in all modern natural products discovery programs, structural novelty should ideally be coupled with the discovery of natural functions and useful biological activity. Incorporation of these new compounds into screening libraries will be limited by the effort needed to grow these fungi and activate their pathways, and by cohesiveness and dedication of the research teams and their abilities to build alliances with drug discovery researchers. Building screening collections of significant value requires a sustained and coordinated effort, but should ultimately lead to the discovery of unique chemistry with varied applications.
